# The associations between bone mineral density and long-term risks of cardiovascular disease, cancer, and all-cause mortality

**DOI:** 10.3389/fendo.2022.938399

**Published:** 2022-09-23

**Authors:** Lin Shi, Xiao Yu, Qingjiang Pang, Xianjun Chen, Chenghao Wang

**Affiliations:** Department of Orthopedics, HwaMei Hospital, University of Chinese Academy of Sciences, Ningbo, China

**Keywords:** bone mineral density, osteoporosis, osteopenia, cardiovascular disease, cancer, all-cause mortality

## Abstract

**Objective:**

We aimed to investigate the associations between bone mineral density and long-term risks of cardiovascular disease (CVD), cancer, and all-cause mortality in nationwide survey participants aged 18 and over.

**Methods:**

Using data from the United States National Health and Nutrition Examination Survey III (NHANES III), the associations of bone mineral density (normal bone mass, osteopenia, and osteoporosis) with CVD, cancer, and all-cause mortality were analyzed using the Cox proportional hazards model.

**Results:**

A total of 11,909 adults aged 18 and over were enrolled in this study. Compared with the participants with normal bone mass, those with osteoporosis and osteopenia were more likely to be female, of non-Hispanic white ethnicity, and older. They were also more likely to have lower calcium and vitamin D intakes, a lower body mass index (BMI), lower educational attainment, and lower family incomes. Participants with osteoporosis and osteopenia also engaged in less physical activity and were more likely to have diabetes, high blood pressure, and a history of CVD. After adjusting for confounders, osteopenia and osteoporosis were significantly associated with all-cause mortality, with the hazard ratios (95% confidence intervals) being 1.37 (1.11, 1.68) and 1.06 (0.91, 1.25), respectively, compared with normal bone mass. Age (P for interaction = 0.001) and BMI (P for interaction = 0.002) were found to modify the association between bone mineral density and all-cause mortality.

**Conclusions:**

In a nationally representative cohort, osteoporosis was associated with an increased risk of all-cause mortality, and this association was stronger in participants who were older and had a lower BMI.

## Introduction

Bone mineral density (BMD) is the bone mineral content in bone tissue and is a measurable indicator related to bone mass and bone strength (1). The World Health Organization (WHO) defines osteoporosis, osteopenia, and normal bone mass according to the T-values of BMD (2, 3). Osteporosis is a systemic skeletal disease that leads to decreased BMD, decreased bone strength, and deterioration of bone microarchitecture, thereby increasing the susceptibility to fragility fractures (4). With the accelerated aging of the population, its incidence increased gradually (4).

A number of epidemiological results have shown that there is a confirmed correlation between osteoporosis and cardiovascular disease (CVD) in both men and women (5). Studies have shown that using BMD as a risk factor for CVD events and death can better predict the development of lesions than traditional risk factors such as hyperlipidemia and smoking (6). Osteoporosis leads to an increased risk of bone fragility and fractures, with clinical hip fractures and vertebral fractures being its most serious consequences (2, 7). However, the associations of reduced BMD with CVD, cancer, and all-cause mortality have not been conclusively established.

Given this context, the aim of the current study was to investigate the associations of BMD with CVD, cancer, and all-cause mortality in a large, nationally representative sample of non-institutionalized adults in the USA.

## Materials and methods

### Study population

The National Health and Nutrition Examination Survey III (NHANES III) was a survey of the health and nutrition status of U.S. residents aged 2 months and older conducted by the National Center for Health Statistics between 1988 and 1994. The survey combined interviews with physical examinations: the interviews included demographic, socioeconomic, dietary, and health-related questions, while the medical examinations included oral examinations, physical measurements, and laboratory tests. The survey protocol was approved annually by the National Center for Health Statistics (NCHS) Research Ethics Review Board, and all participants provided written informed consent. The details of the survey design and collection procedures have been reported previously (8).

**Figure 1:**
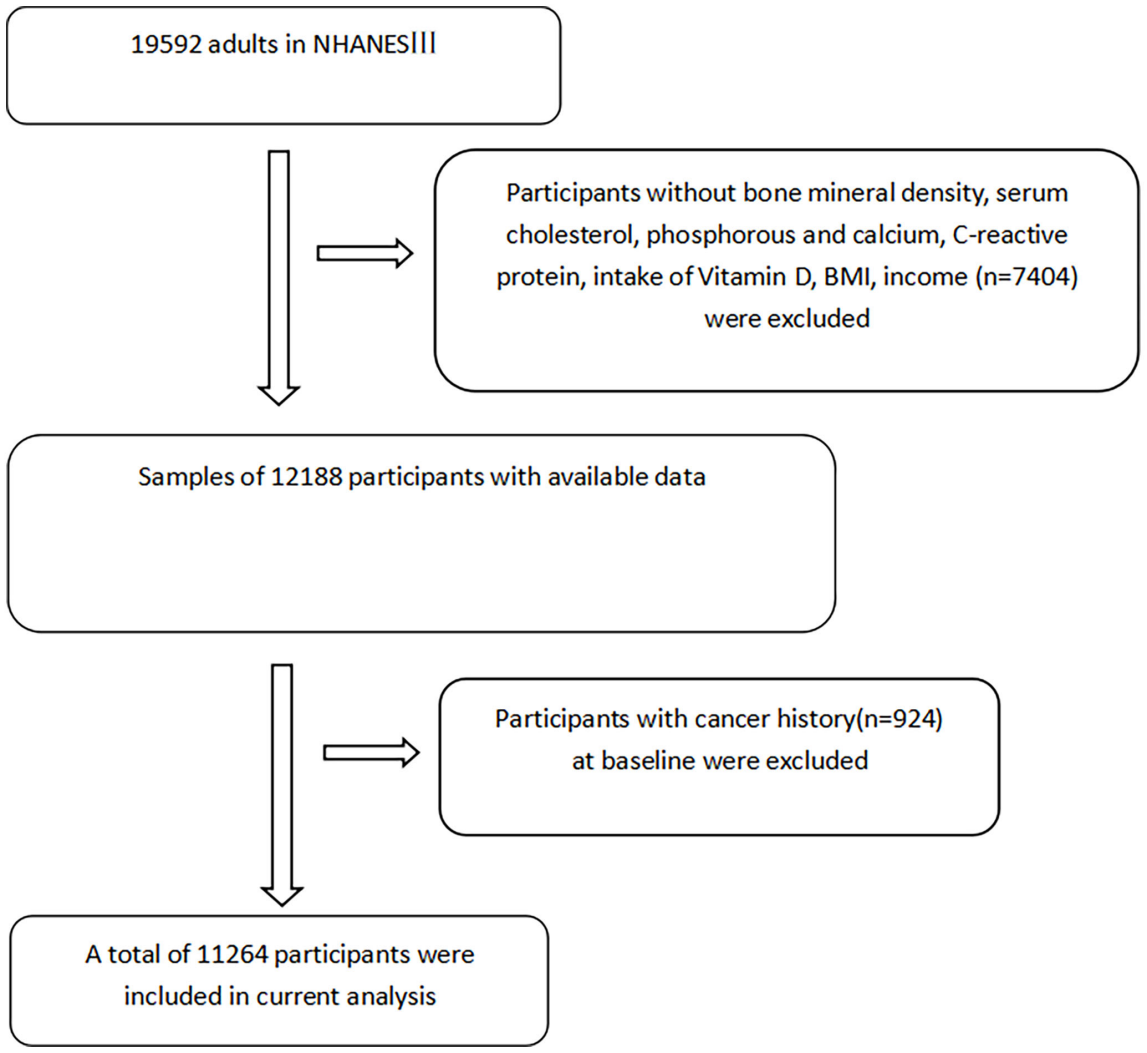
The flowchart of included participants.

A total of 19,592 participants aged 18 years and older were selected from NHANES III for this study. Participants aged below 18 years and younger were excluded in our study, because we restricted our analysis to only adults. Those with missing data relating to BMD, vitamin D, and history of CVD and cancer were excluded. The flowchart for the selection of study participants is shown in **Figure 1**.

### Osteoporosis

The femur T-score was used to evaluate bone health. Bone health was classified as normal, osteopenic, or osteoporotic based on the T-scores (≥-1.0, <- 1 to >- 2.5, and ≤-2.5,respectively) as recommended by the WHO (9). Young female adult aged from 20 to 29 years in average were used as the reference group for T-score calculation at this study (10).

### Outcome assessment

The follow-up data on all-cause, CVD, and cancer mortality were gathered primarily from mortality documents published by the NCHS, as the mortality outcomes of the study participants was recorded until December 31, 2015. All of the causes of death were recorded according to the classification system of the International Classification of Diseases, 10^th^ Edition (ICD-10). CVD and cancer deaths were classified using the ICD-10 codes I00–I78 and C00–C97, respectively.

### Covariate assessment

Standardized questionnaires were used to obtain information on the following participant characteristics: age, sex, calcium intake, vitamin D intake, body mass index (BMI), race (non-Hispanic white, non-Hispanic black, Mexican American, others), educational attainment (some high school or less, high school graduate, above high school level), and annual household income (< $20,000, $20,000–$26,000, > $26,000), physical activity (poor, intermediate, ideal) (11), alcohol intake (never, 0–18 mg, > 18 mg), smoking status (never, former, current), hypertension, diabetes, and history of CVD and cancer.

Because NHANES III did not ask the duration of physical activities, we defined physically active as participants as ideal if they engaged in physical activities with 3≤metabolic equivalents<6 and ≥5 times/wk or physical activities with metabolic equivalents ≥6 and 3.0 times/wk. Physical activities included walking, jogging or running, bicycling, swimming, aerobics or aerobic dancing, other dancing, calisthenics, gardening or yard work, and other sports. The difference between physically active and no physical activity was taken as intermediate. Those who didn’t take any physical activities were defined as poor (11).

CVD was defined as the composite self-reported congestive heart failure, coronary heart disease, angina/angina pectoris, heart attack, or stroke. Hypertension was defined as a mean SBP ≥130 mmHg, or a mean DBP≥80 mmHg, or a self-reported antihypertension medication use currently, according to the 2017 ACC/AHA Guideline for High Blood Pressure in adults (12). Diabetes was defined as self-report diabetes (Participants answering “yes” to the question, “Doctor told you have diabetes”) or current use of hypoglycemic agents or a hemoglobin A_1c_ (HbA_1c_) level ≥ 6.5% (13).

### Statistical analysis

All of the participants were classified according to their T-score into three groups: normal bone mass, osteopenia, and osteoporosis. The NHANES used a complex design. Weight was taken into consideration. All data were analyzed based on weighted estimates with sample weights provided by NHANES. Continuous variables are shown as weighted mean and SE, while categorized variables are described as frequency and weighted percentage.

The qualitative variables were compared using a chi-square test and were reported as frequencies and proportions. The quantitative variables were compared using the non-parametric test and was reported as medians. The linear trends in baseline characteristics across the BMD groups were assessed using an analysis of covariance for continuous variables and a Cochran–Armitage trend test for categorical variables.

Multivariate Cox proportional risk models were used to assess the relationships between BMD (normal bone mass, osteopenia, and osteoporosis) and all-cause, CVD, and cancer mortality with the following covariates: age and gender (model 1); model 1 plus BMI, race, education, income, physical activity, alcohol intake, smoking status, hypertension, diabetes, history of CVD, and history of cancer (model 2); and model 2 plus vitamin D intake and serum cholesterol, phosphorus, calcium, and C-reactive protein (model 3). Proportional hazards assumptions were assessed by analyzing Schoenfeld residuals.

Stratified analyses were performed to assess the potential modifying effects of the following variables on the association between BMD and mortality outcomes: age (< 50 or ≥ 50 years), sex (male or female), BMI (< 25 or ≥ 25 kg/m^2)^, and race (non-Hispanic white, non-Hispanic black, Mexican American, others). Interactions were analyzed using the likelihood ratio test with and without the cross-product interaction term and by adjusting for the variables in model 3. We excluded outcome events that occurred within the 2-year follow-up period, to assess whether the results had been influenced by reverse causation.

All of the statistical analyses were conducted using survey modules in SAS software, version 9.4 (SAS Institute, Cary, NC, USA). Two-sided P-values < 0.05 were regarded as statistically significant.

## Results

### Baseline characteristics

A total of 11,264 participants aged 18 and above were included in our analysis: 467 cases of osteoporosis, 4,113 cases of osteopenia, and 6,684 cases of normal bone mass (**Figure 1**; **Table 1**). **Table 1** shows the baseline data of participants with osteoporosis, osteopenia, and normal bone mass. Compared with the participants with normal bone mass, those with osteoporosis and osteopenia were more likely to be female, of non-Hispanic white ethnicity, and older. They were also more likely to have lower calcium and vitamin D intakes, a lower BMI, less than a high school education, and lower family incomes. They also engaged in less physical activity and were more likely to have diabetes, high blood pressure, and a history of CVD.

**Table 1:** Baseline characteristics according to the groups of bone mineral density(mg/cm2)^a^.

Variables	Normal	Osteopenia	Osteoporosis	*P*-trend
N	6684	4113	467	
age(years)^bcd^	36	55	75	< 0.0001
serum total calcium(mg/dL)	9.30	9.30	9.30	0.1736
vitamin D(μg)^c^	3.10	3.40	3.90	0.9760
serum cholesterol(mmol/L)^bcd^	5.02	5.40	5.77	< 0.0001
serum phosphorus (mg/dL)^b^	3.50	3.40	3.50	0.0470
serum C-reactive protein	0.21	0.21	0.21	0.0133
BMI(kg/m^2^)^bcd^	27.20	25.30	23.20	< 0.0001
gender(%)^bcd^				< 0.0001
male	3371(56.42)	1663 (40.43)	82 (17.56)	
female	2913(43.58)	2450 (59.57)	385 (82.44)	
race(%)^bcd^				< 0.0001
non-Hispanic white	2025 (30.30)	2197 (53.42)	347 (74.30)	
non-Hispanic black	2380 (35.61)	716 (17.41)	37 (7.92)	
mexican American	2032 (30.40)	1004 (24.41)	62 (13.28)	
others	247 (3.70)	196 (4.77)	21 (4.50)	
education(%)^bcd^				< 0.0001
less than high school	2414 (36.12)	1677 (40.77)	225 (48.18)	
high school graduate	3395 (50.79)	1861 (45.25)	197 (42.18)	
great than high school	875 (13.09)	575 (13.98)	45 (9.64)	
income(%)^bcd^				0.4490
<20000	3051 (45.79)	1871 (45.55)	276 (59.23)	
20000~26000	2697 (40.48)	1580 (38.46)	151 (32.40)	
>26000	915 (13.73)	657 (15.99)	39 (8.37)	
physical activity(%)^bcd^				< 0.0001
poor	1186 (17.74)	891 (21.66)	162 (34.69)	
intermediate	3452 (51.65)	2022(49.16)	168 (35.97)	
ideal	2046 (30.61)	1200 (29.18)	137 (29.34)	
alcohol(%)^bcd^				< 0.0001
never	5051 (75.57)	3348 (81.4)	426 (91.22)	
0~18mg	483 (7.23)	275 (6.69)	24 (5.14)	
>18mg	1150 (17.30)	490 (11.91)	17 (3.64)	
tobacco(%)^bcd^				< 0.0001
never	3150 (47.13)	1843 (44.81)	278 (59.53)	
former	1490 (22.29)	1191 (28.96)	113 (24.20)	
current	2044 (30.58)	1079 (26.23)	76 (16.27)	
hypertension(%)^bcd^				< 0.0001
NO	5524 (78.16)	2646 (64.33)	189 (40.47)	
YES	1460 (21.84)	1467 (35.67)	278 (59.53)	
diabetes(%)^cd^				< 0.0001
NO	6140 (91.86)	3667 (89.16)	411 (88.01)	
YES	544 (8.14)	446 (10.84)	56 (11.09)	
CVDhistory(%)^bcd^				< 0.0001
NO	6394 (95.66)	3723 (90.52)	390 (83.51)	
YES	290 (4.34)	390 (9.48)	77 (16.49)	

BMD, Bone mineral density(mg/cm^2^).

a The NHANES used a complex design. Weight was taken into consideration. Continuous variables are shown as weighted mean and SE, while categorized variables are described as frequency and weighted percentage. All data were analyzed based on weighted estimates with sample weights provided by NHANES.

b normal vs. osteopenia, P < 0.01.

c normal vs. osteoporosis, P < 0.01.

d osteopenia vs. osteoporosis, P < 0.01.

Non-parametric test was used to compare the median levels of continuous variables and chi-square tests were used to compare the distribution of categorical variables.

### Long-term follow-up

**Figure 2:**
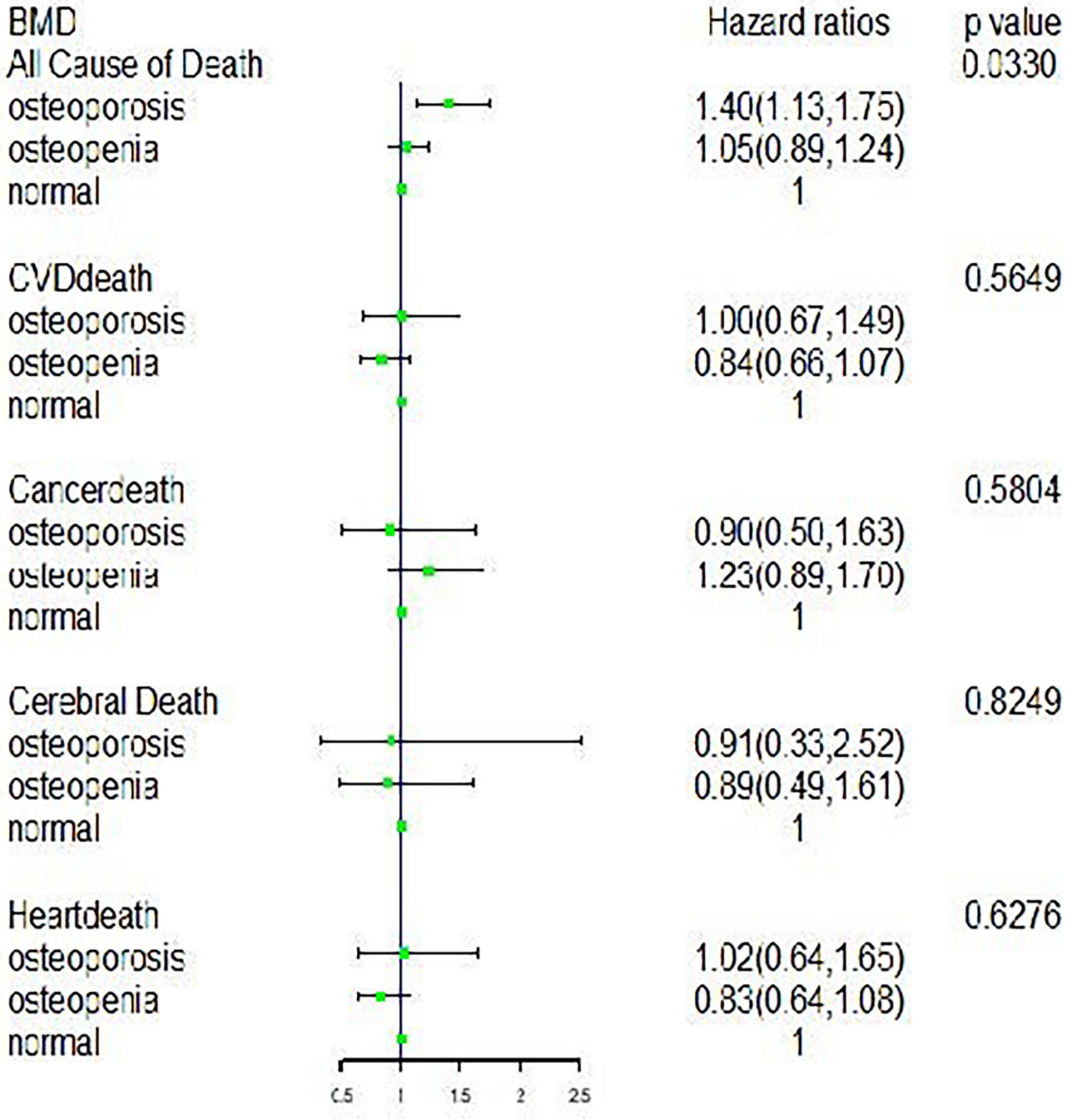
The forest plot about hazard ratios (95% CI) for CVD, cancer and all-cause mortality.

Across the 195,857 person-years of follow-up, we identified 3,110 deaths, including 974 from CVD, 217 from cerebral mortality, 757 from heart disease mortality, and 657 from cancer. The associations of BMD with all-cause mortality, CVD mortality, cancer mortality, cerebral mortality, and heart mortality are shown in **Table 2** and **Figure 2**. We observed a statistically significant association between BMD and all-cause mortality: osteopenia and osteoporosis were significantly associated with all-cause mortality, with the mortality hazard ratio (HR) (95% confidence intervals [CIs]) being 1.37 (1.11, 1.68) and 1.06 (0.91, 1.25), respectively, compared with normal bone mass in model 3.

**Table 2:** Hazard Ratios (95% CI) for Risk of CVD, cancer and all-cause mortality according to the groups of bone mineral density.

	Normal	Osteopenia		Osteoporosis			Each SD	
		Hazards ratio	95CI	Hazards ratio	95CI	*P*-trend	Hazards ratio	95CI
Median(gm/cm^2^)	0.93	0.72		0.51				
All-cause mortality								
N/person-years	1167/123,669	1580/66,982		363/5207				
Model 1	1.00	0.97	0.85,1.11	1.17	0.98,1.40	0.3482	0.85	0.60,1.21
Model 2	1.00	1.07	0.90,1.26	1.40	1.12,1.76	0.0308	0.56	0.32,1.00
Model 3	1.00	1.05	0.89,1.24	1.40	1.13,1.75	0.0330	0.58	0.32,1.04
CVD mortality								
N/person-years	323/123,669	531/66,982		120/5207				
Model 1	1.00	0.80	0.64,1.00	0.80	0.52,1.22	0.1609	1.42	0.59,3.41
Model 2	1.00	0.87	0.69,1.10	0.99	0.66,1.48	0.6400	0.87	0.34,2.24
Model 3	1.00	0.84	0.66,1.07	1.00	0.67,1.49	0.5649	0.93	0.36,2.42
Cancer mortality								
N/person-years	277/123,669	344/66,982		36/5207				
Model 1	1.00	1.18	0.86,1.61	0.78	0.43,1.42	0.9229	1.07	0.43,2.66
Model 2	1.00	1.25	0.91,1.72	0.90	0.51,1.60	0.5391	0.79	0.26,2.40
Model 3	1.00	1.23	0.89,1.70	0.90	0.50,1.63	0.5804	0.83	0.27,2.57
Cerebral mortality								
N/person-years	70/123,669	120/66,982		27/5207				
Model 1	1.00	0.82	0.48,1.42	0.76	0.31,1.85	0.5068	1.34	0.19,9.60
Model 2	1.00	0.90	0.51,1.61	0.89	0.32,2.48	0.8019	0.80	0.07,8.83
Model 3	1.00	0.89	0.49,1.61	0.91	0.33,2.52	0.8249	0.80	0.07,9.19
Heart disease mortality								
N/person-years	253/123,669	411/66,982		93/5207				
Model 1	1.00	0.79	0.63,1.00	0.81	0.49,1.34	0.2124	1.45	0.57,3.73
Model 2	1.00	0.86	0.66,1.12	1.03	0.64,1.64	0.7059	0.90	0.30,2.66
Model 3	1.00	0.83	0.64,1.08	1.02	0.64,1.65	0.6276	0.97	0.33,2.89

Model1: adjusted for age, gender.

Model2: including model 1 and adjusted for intake of vitamin D, serum cholesterol, BMI, race, education, income, physical activity, alcohol intake, smoking status, hypertension, diabetes, history of CVD and cancer.

Model3: including model 2 and adjusted for serum cholesterol, phosphorus and calcium, C-reactive protein.

### Subgroup analyses

We conducted several stratified analyses. After adjusting for potential risk factors, we found that the association between BMD and all-cause mortality was modified by age. Specifically, osteoporosis was associated with an increased risk of all-cause mortality in older participants: the HR for participants aged 50 years and over was 2.80 (95% CI: 2.29, 3.42; P for interaction = 0.001; see **Table 3**). Osteoporosis was also associated with an increased risk of all-cause mortality in participants with a lower BMI: the HR for participants with a lower BMI was 1.49 (95% CI: 1.08, 2.07; P for interaction = 0.001, **Table 3**).

**Table 3:** Subgroup analysis for CVD, cancer and all-cause mortality according to the groups of bone mineral density (mg/cm2).

All-cause mortality		Osteoporosis	Osteopenia	Normal	*P*-trend	*P*-interaction
Age	age<50 years	2.62 (0.25,27.64)	1.40 (0.99,1.98)	1	0.0382	0.0017
	age≥50 years	2.80 (2.29,3.42)	1.43 (1.20,1.71)	1	< 0.0001	
Gender	male	1.00 (0.09,11.25)	1.00 (0.82,1.22)	1	1	0.4129
	female	1.35 (1.03,1.77)	1.08 (0.84,1.39)	1	0.0257	
Race	non-Hispanic white	1.42 (1.09,1.85)	1.05 (0.86,1.21)	1	0.0547	0.1302
	non-Hispanic black	1.34 (0.91,1.98)	1.05 (0.86,1.27)	1	0.3182	
	mexican American	1.32 (0.85,2.05)	1.25 (1.00,1.57)	1	0.0353	
	other	0.73 (0.25,2.15)	0.92 (0.45,1.89)	1	0.6313	
BMI	BMI<25 kg/m^2^	1.49 (1.08,2.07)	1.11 (0.81,1.53)	1	0.0149	0.0025
	BMI≥25 kg/m^2^	1.20 (0.85,1.69)	1.00 (0.84,1.20)	1	0.5817	
**CVD mortality**						
Age	age<50 years	0	1.40 (0.99,1.98)	1	0.6875	0.0821
	age≥50 years	2.30 (1.57,3.35)	1.27 (0.96,1.67)	1	0.0008	
Gender	male	1.30 (0.62,2.70)	0.80 (0.58,1.10)	1	0.3943	0.1679
	female	0.99 (0.63,1.56)	0.94 (0.67,1.30)	1	0.9804	
Race	non-Hispanic white	0.95 (0.61,1.49)	0.80 (0.61,1.06)	1	0.5065	0.4231
	non-Hispanic black	1.07 (0.53,2.17)	1.30 (0.88,1.93)	1	0.2871	
	mexican American	2.17 (0.83,5.65)	1.40 (0.97,2.04)	1	0.0483	
	other	0.28 (0.02,3.87)	0.20 (0.07,0.56)	1	0.0162	
BMI	BMI<25 kg/m^2^	1.38 (0.42,4.50)	0.62 (0.15,2.53)	1	0.4649	0.1292
	BMI≥25 kg/m^2^	1.01 (0.55,1.86)	0.89 (0.67,1.19)	1	0.6727	
**Cancer mortality**						
Age	age<50 years	0	2.34 (1.42,3.86)	1	0.0015	0.075
	age≥50 years	1.33 (0.75,2.34)	1.25 (0.91,1.72)	1	0.1403	
Gender	male	0.53 (0.12,2.41)	1.25 (0.82,1.91)	1	0.5357	0.1122
	female	1.18 (0.57,2.45)	1.29 (0.79,2.09)	1	0.5054	
Race	non-Hispanic white	1.04 (0.52,2.07)	1.31 (0.89,1.93)	1	0.4634	0.421
	non-Hispanic black	0.51 (0.11,2.35)	0.85 (0.58,1.24)	1	0.1775	
	mexican American	0.13 (0.02,0.75)	1.57 (0.70,3.53)	1	0.7119	
	other	0.20 (0.02,2.00)	1.21 (0.31,4.68)	1	0.6598	
BMI	BMI<25 kg/m^2^	1.46 (0.60,3.59)	1.98 (1.07,3.69)	1	0.1818	0.3094
	BMI≥25kg/m^2^	0.64 (0.25,1.64)	0.98 (0.65,1.46)	1	0.6401	
**Cerebral mortality**						
Age	age<50 years	0	0.85 (0.13,5.71)	1	0.8442	0.1441
	age≥50 years	2.75 (0.97,7.78)	1.54 (0.78,3.04)	1	0.0657	
Gender	male	2.99 (0.53,16.80)	099 (0.39,2.48)	1	0.6714	0.9782
	female	0.66 (0.19,2.24)	0.81 (0.36,1.82)	1	0.4954	
Race	non-Hispanic white	1.00 (0.28,3.56)	0.99 (0.42,2.39)	1	0.9996	0.0374
	non-Hispanic black	0.74 (0.14,3.87)	1.28 (0.65,2.51)	1	0.7815	
	mexican American	0.33 (0.05,2.02)	0.73 (0.30,1.81)	1	0.3020	
	other	0	2.43 (0.04,146.33)	1	< 0.0001	
BMI	BMI<25 kg/m^2^	2.49 (0.06,99.42)	0.50 (0.02,15.92)	1	0.3836	0.5789
	BMI≥25kg/m^2^	1.39 (0.45,4.30)	1.16 (0.57,2.37)	1	0.575	
**Heart disease mortality**						
Age	age<50	0	0.87 (0.33,2.33)	1	0.7651	0.18
	age≥50	2.22 (1.38,3.55)	1.21 (0.90,1.63)	1	0.0099	
Gender	male	1.10 (0.52,2.36)	0.77 (0.55,1.08)	1	0.2501	0.1169
	female	1.16 (0.64,2.12)	0.99 (0.66,1.49)	1	0.5961	
Race	non-Hispanic white	0.97 (0.59,1.62)	0.79 (0.58,1.62)	1	0.4832	0.701
	non-Hispanic black	1.20 (0.43,3.34)	1.32 (0.79,2.20)	1	0.3673	
	mexican American	4.03 (1.47,11.04)	1.85 (1.25,2.74)	1	0.0017	
	other	0.72 (0.07,7.06)	0.39 (0.14,1.14)	1	0.3418	
BMI	BMI<25 kg/m^2^	0.84 (0.40,1.75)	0.72 (0.37,1.40)	1	0.6573	0.0826
	BMI≥25 kg/m^2^	0.95 (0.47,1.91)	0.84 (0.62,1.14)	1	0.473	

Adjusted for variables in model 4.

### Sensitivity analysis

To examine the role of reverse causation, 212 participants whose outcomes occurred during the first 2 years of follow-up were excluded from the analysis. The results obtained using the remaining sample were similar to those observed in the full sample (data not shown). We further adjusted for COPD, and the results did not change significantly (data not shown).

## Discussion

In this large prospective study of a nationally representative cohort, we found that, after multivariate adjustment, osteoporosis and osteopenia were independently associated with an increased risk of all-cause mortality at long-term follow-up. These associations were stronger in participants who were older and those who had a lower BMI. These trends remained robust in stratified and sensitivity analyses.

One study performed in US participants found a higher risk of all-cause mortality among participants with osteoporosis compared with normal in the regions of total femur, femur neck, intertrochanter, as well as overall (14). Michael et al. found that subjects in the lowest quartile of BMD in total femur showed a 53% increase in all-cause mortality, in contrast to those in the highest quartile, in U.S. population aged 50 years and older (15). Also, low BMD is a strong and independent predictor of all-cause and CVD mortality, particularly in men (16).

Our study demonstrated that osteoporosis is associated with an increased risk of all-cause mortality. A similar prospective cohort study in the United Kingdom also showed that patients with osteoporosis had a higher risk of all-cause mortality, as well as higher CVD and cancer mortality, than controls (17). In a gender-disaggregated analysis, men with osteoporosis were shown to have higher rates of all-cause mortality, respiratory diseases, including chronic obstructive pulmonary disease, and cancer than women (17). McFarlane et al. also found that patients with osteopenia had higher cardiovascular mortality than individuals with normal bone mass (18). People who were older, thinner, and had a lower BMI had a higher risk of developing osteoporosis (19). Notably, non-significant results were observed for osteoporosis with CVD, and cancer mortality in our study, the non-significant results might be due to the lower number of incident CVD, and cancer deaths, and short-time follow-up.

We also compared the baseline data of participants with normal bone mass, osteopenia, and osteoporosis. The results showed that compared with the participants with normal bone mass, those with osteoporosis and osteopenia were older and more likely to be female. This may be related to bone loss due to aging or menopause (20). Non-Hispanic whites and Mexican Americans had a higher risk of osteopenia and osteoporosis than non-Hispanic blacks, which is consistent with previous findings regarding the prevalence of osteoporosis and osteopenia in the United States (21). Studies have shown that osteopenia and osteoporosis are associated with lower serum calcium levels than normal bone mass (22, 23). The results of a randomized controlled trial involving Japanese women showed that increasing their daily calcium intake helped to reduce bone loss in the lumbar spine, an effect that was related to the calcium dose (24).

The difference in vitamin D intake among the three BMD groups in our study was also statistically significant, with vitamin D intake being lower in the osteopenia and osteoporosis groups than in the normal bone mass group. Multiple studies have shown that vitamin D supplementation can significantly improve bone mineral in postmenopausal women (8, 25, 26). Studies have also shown that compared with the BMI of people with osteoporosis and osteopenia, that of people with normal bone mass is relatively higher, and there is evidence that BMD is positively correlated with BMI (27). There were also statistical differences in family income and education level among the three BMD groups. This is consistent with previous findings that family income and education level affect the degree to which osteoporosis is successfully prevented (28).

Physical activity can improve or maintain BMD in people of all ages. Studies have shown that exercise can improve the physical function and quality of life of postmenopausal women with osteoporosis and osteopenia, with high-intensity resistance training being particularly useful for increasing BMD and preventing osteoporosis (29). In our study, diabetes was found to be associated with increased risks of osteopenia and osteoporosis. This is consistent with findings based on the Danish National Health Survey (30). Studies have shown that patients with a history of osteoporotic fractures or low BMD have higher risks of coronary artery disease and stroke than those without osteoporosis, while patients with CVD have higher risks of bone loss and osteoporotic fractures than those without CVD (31). This is exactly in line with our findings.

The results of both cross-sectional studies and case–control studies show that hypertension is negatively correlated with BMD (32) and that postmenopausal women with hypertension have a higher risk of osteoporosis than those without hypertension (33). Studies have shown that drinking alcohol is positively correlated with the occurrence of osteoporosis (34–36). A study in Taiwan showed that smoking is a risk factor for osteoporosis in men but not in women, and the association observed in men may be diluted by bone loss due to menopause in women (37). However, we did not find a consistent association of smoking and alcohol consumption with osteoporosis, so further research is needed.

To the best of our knowledge, this is the first large prospective study to assess the associations of BMD with the risks of all-cause mortality, CVD mortality, and cancer mortality in a general US population sample. One strength of our study is its large, nationally representative cohort design, which facilitates the generalization of our findings to the general US population. Another strength is its use of the data collected using standardized procedures, with quality control measures and adjustments to control for potentially confounding effects. We found that the associations of BMD were robust to the exclusion of subjects who died within the first 2 years of follow-up.

However, our study also has several limitations. First, the data on household income, physical activity levels, smoking history, and dietary intakes were self-reported by participants in NHANES III and may therefore be subject to reporting bias. Second, there may have been misclassifications of the underlying and contributing causes of death and residual confounding and competing risks for cause-specific mortalities. In addition, although we adjusted for the common risk factors known to be associated with mortality, some uncontrolled and unmeasured confounders might have remained.

In conclusion, our study findings suggest osteoporosis and osteopenia were independently associated with an increased risk of all-cause mortality at long-term follow-up after multivariate adjustment. These associations were stronger in participants who were older and those who had a lower BMI. Prospective studies examining other populations are needed to confirm our findings. Future studies examine the correlation of the ten-year fracture risk (FRAX index) with the risks of all-cause mortality, CVD mortality, and cancer mortality are also necessary.

## Data availability statement

The raw data supporting the conclusions of this article will be made available by the authors, without undue reservation.

## Ethics statement

The survey protocol was approved annually by the National Center for Health Statistics (NCHS) Research Ethics Review Board, and all participants provided written informed consent.

## Author contributions

XY conceived the idea for this initiative. LS and QP contributed to reading the literature, preparation of figures and the table, and writing the manuscript; XC and CW assisted with writing and revising the manuscript. All authors read and approved the final manuscript.

## Funding

This work was supported by Zhejiang Medical and Health Science and Technology Project (2022ky329,2022ky1129); Zhejiang Traditional Medicine and Technology Project (2020ZB227); Ningbo Science and Technology Innovation 2025 Specific Project (2020Z096) and Ningbo Medical & health Leading Academic Discipline Project(2022-F15).

## Acknowledgments

We thank all authors for their contributions to the article.

## Conflict of interest

The authors declare that the research was conducted in the absence of any commercial or financial relationships that could be construed as a potential conflict of interest.

## Publisher’s note

All claims expressed in this article are solely those of the authors and do not necessarily represent those of their affiliated organizations, or those of the publisher, the editors and the reviewers. Any product that may be evaluated in this article, or claim that may be made by its manufacturer, is not guaranteed or endorsed by the publisher.
